# A Survey-Based Assessment of Awareness Regarding Parthenium hysterophorus in a Rural Population of North India

**DOI:** 10.7759/cureus.41453

**Published:** 2023-07-06

**Authors:** Satyendra K Sharma, Siddhartha Dash, Prakriti Shukla, Riya Gupta, Uzma Sami

**Affiliations:** 1 Dermatology, Venereology and Leprosy, Hind Institute of Medical Sciences, Sitapur, IND; 2 Dermatology and Venereology, Srirama Chandra Bhanja Medical College and Hospital, Cuttack, IND

**Keywords:** rural india, awareness survey, congress grass, scourge of india, parthenium hysterophorus

## Abstract

Background

*Parthenium hysterophorus* is a poisonous weed that has spread across the length and breadth of the Indian subcontinent. It is a common cause of dermatitis similar to other members of the family, such as ragweeds in the United States and chrysanthemums in Europe. Despite the common occurrence of the plant in the environment, the general population does not seem to be aware of its adverse effects. This cross-sectional study was conducted with the aim of assessing the awareness of *P. hysterophorus* in the local rural population.

Methodology

A questionnaire was designed by an expert panel consisting of three dermatologists after a bibliological survey and collection of published literature on *P. hysterophorus*. All adult patients >18 years of age approaching the outpatient department of dermatology at our tertiary health care center were included in a consecutive sampling manner. Patients with an unsound mind or those who refused to provide consent were excluded from the study.

Results

A total of 250 patients participated in the study, of whom 56.8% were male and 43.2% were female, with ages ranging from 18 to 80 years. The majority of the participants were farmers by occupation. Three-fourths of the participants (187, 74.8%) were able to identify the plant. Maximum participants (144, 57.6%) did not know about the ill effects of *Parthenium*, and 148 (59.2%) did not know about the method of controlling its spread.

Conclusions

The study was conducted among 250 respondents from a rural community in North India. Despite almost 75% of participants identifying the weed, more than half were neither aware of its ill effects nor knew about the methods of control. Furthermore, most participants were educated individuals yet remained ignorant. Emphasis needs to be made on awareness campaigns about the fast-spreading *Parthenium *and its ill effects.

## Introduction

*Parthenium hysterophorus*, commonly known as “Congress grass” or the “scourge of India,” is a poisonous weed that has spread across the length and breadth of the Indian subcontinent. Belonging to the family Compositae (now known as Asteraceae), it is a common cause of dermatitis similar to other members of the family, such as ragweeds in the United States and chrysanthemums in Europe [1]. Other than causing contact dermatitis, it is also responsible for incidences of asthma, hay fever, and bronchitis, as well as causing harm to the local flora and fauna [1]. Despite the common occurrence of the plant in the environment, the general population does not seem to be aware of its adverse effects. This cross-sectional study was conducted with the aim of assessing the awareness of *P. hysterophorus* in the local rural population, and thereby identify steps that can be taken to curb its ill effects.

## Materials and methods

The study was conducted at a tertiary care institute for six months. Institutional ethical clearance was obtained before the commencement of the study (IEC/IRB number: HIMS/IRB/2020-21/45). At the onset of the study, a questionnaire was designed by an expert panel consisting of three dermatologists (SS, SD, PS) that performed a bibliological survey and collected published literature on *P. hysterophorus*. A literature review on the health hazards of *P. hysterophorus* was done, as well as to identify the existing data on its awareness among the rural population. This was followed by the generation of a preliminary question pool that later formed the basis for the questionnaire used in the study.

All adult patients >18 years of age approaching the outpatient department of dermatology at our tertiary health care center were included after obtaining written informed consent in a consecutive sampling manner. Patients with an unsound mind or those who refused consent were excluded from the study. Sociodemographic details and education status were recorded in a predesigned proforma in addition to the questionnaire. Moreover, the local terminology for the *Parthenium *weed was noted in the proforma.

## Results

A total of 250 patients participated in the study, of whom 142 (56.8%) were male, and 108 (43.2%) were female. The minimum age was 18 years, and the age of the oldest participant was 80 years. The mean age of the participants was 38.99 ± 14.62 years, and more than half of them were in the age group of 18-39 years. The majority of the participants were farmers (107, 42.8%) by occupation (Table 1).

**Table 1:** Sociodemographic variables of the study participants (N = 250).

Variable	Categories	Frequency (%)
Age (mean ± SD)		38.99 ± 14.62
Age group (years)	18–39	132 (52.8)
	40–59	89 (35.6)
	≥60	29 (11.6)
Gender	Male	142 (56.8)
	Female	108 (43.2)
Occupation	Farmer	107 (42.8)
	Housewife	78 (31.2)
	Student	33 (13.2)
	Teacher	8 (3.2)
	Driver	7 (2.8)
	Tailor	7 (2.8)
	Other	7 (2.8)
Educational qualification	Illiterate	67 (26.8)
	Primary	48 (19.2)
	High school	27 (10.8)
	Intermediate	40 (16.0)
	Graduate	30 (12.0)
	Postgraduate	17 (6.8)
	Middle school	21 (8.4)
Substance use	No addiction	167 (66.8)
	Smokeless tobacco	37 (14.8)
	Smoking tobacco	47 (18.8)
	Alcohol	30 (12.0)
Median family size (median, IQR)		5 (4-8)
Mean family income (median, IQR) in INR		6,000 (5,000–10,000)

Parthenium was known by various names in the local rural population, of which the most common were “Phulera/philora,” “gajar ghaas,” “angrezi ghaas,” “jungli ghass,” and “nayi ghaas.”

Only three-fourths of the participants (187, 74.8%) responded that they can identify the plant. The majority of the participants (115, 46.0%) knew about it as they had seen it in the surrounding. Most participants (98, 39.2%) did not know about the method of dissemination. One-third of the study participants did not know about the invasion period. The most common ill effect mentioned by the study participants was human diseases (50, 20%), followed by poisonous to animals (23, 9.2%), and 15 (6.0%) mentioned that it affects crop growth. Furthermore, the maximum number of participants (144, 57.6%) did not know about the ill effects of *Parthenium*. The majority of the study participants (148, 59.2%) did not know about the method of controlling its spread (Table 2).

**Table 2:** Knowledge regarding Parthenium among the study participants (N = 250).

Variable	Categories	Frequency (%)
Identify the plant	Yes	187 (74.8)
	No	63 (25.2)
Source of information	Seen in the surrounding	115 (46.0)
	Relatives	54 (21.6)
	Media	18 (7.2)
	Nothing	63 (25.2)
Where have you seen the plant?	Roadside	91 (36.4)
	Wasteland	66 (26.4)
	Railway tract	19 (7.6)
	Nearby	9 (3.6)
	Not seen	65 (26.0)
Near your house	Yes	102 (40.8)
	No	82 (32.8)
	Not known	66 (26.4)
What do you know about dissemination?	Wind	74 (29.6)
	Seed	39 (15.6)
	Water	34 (13.6)
	Animal	5 (2.0)
	Not known	98 (39.2)
Period of invasion	Not known	83 (33.2)
	1–10 years	83 (33.2)
	11–20 years	60 (24.0)
	>20 years	24 (9.6)
What are the ill effects?	Not known	144 (57.6)
	Poisonous to animal	23 (9.2)
	Human disease	50 (20.0)
	Both	18 (7.2)
	Affects crop growth	15 (6.0)
Most affected part of the body	Not known	148 (59.2)
	Skin	67 (26.8)
	Difficulty in breathing	27 (10.8)
	Eyes	8 (3.2)
Method to control the spread	Not known	148 (59.2)
	Manual	38 (15.2)
	Chemical	52 (20.8)
	Biological	12 (4.8)

## Discussion

*P. hysterophorus *is a noxious ubiquitous plant that has rightly achieved the status of one of the most hazardous weeds. A native of Northeast Mexico, it was probably introduced in India along with wheat grains under the PL 480 scheme [2]. The huge seed production ability (10,000-15,000 seeds/plant) and small and light-weight seeds capable of long-distance travel through wind, water, birds, and other animals confer it the ability for wide dissemination. Having an irritating odor, taste, and trichome hairs, livestock find it unpalatable, and consumption causes gastrointestinal irritation or reduced milk production. Furthermore, it also inhibits the growth of other plants due to allelopathic effects. It has also been attributed to various allergies in humans such as contact dermatitis, hay fever, asthma, and bronchitis. The sesquiterpene lactones such as parthenin and cornopilin present in the trichomes of leaves and stems are the most common allergens and play a key role in the above-mentioned allergic conditions [3]. The healthcare burden caused by *Parthenium *can be estimated by the fact that in the dermatology clinics alone, *P. hysterophorus* allergic contact dermatitis contributes to almost 40% of all contact dermatitis cases [4]. The most common presentation is in the form of airborne contact dermatitis, followed by erythroderma and chronic actinic dermatitis [4]. The remaining patients manifest a mixture of these patterns.

Given the associated hazards, it becomes necessary to get rid of the weed through physical, chemical, or biological methods. The physical methods of control involve manual removal by hand weeding or plowing and burning [3]. This is the most cost-effective method but can be time-consuming and carries the risk of allergic reactions on exposure. Synthetic herbicides such as glyphosate can be used for chemical control, but usage in large quantities could be expensive, as well as hazardous for the crops [3]. Biological methods through allelopathic plants, insects, or microbial pathogens provide an environmentally responsible as well as economically viable method of control of *Parthenium *[3]. Biocontrol techniques are promising means to get rid of this noxious weed but these are still under development and there remains some time before these can be released for large-scale use. Another method of control that is being explored is control through utilization where studies are being conducted to search for the utilization potential of this weed. Development is underway with variable success to seek its utilization as a raw material or as a component in herbicides, pesticides, insecticides, ethanol production, composting, green manure, nanoparticle synthesis, silkworm feed additives, soaps, and de-colorizing agents [5-11].

Few studies have investigated the awareness regarding the ill effects of *Parthenium *among the population. As our hospital is one of the premier tertiary health care centers and is located in rural premises, it attracts much of the local rural population for their needs. This allowed us to access the data in our target population, that is, the rural community. Although farmers are more exposed to the *Parthenium *weed due to their occupation, the entire rural population is at risk of developing one or the other health hazards associated with *Parthenium*. Most previous studies have been performed keeping the farmer population in mind. In our study, more than 42% of the participants were farmers by occupation, and though almost 75% of them could identify the *Parthenium *weed, more than half of the participants did not know about its ill effects. This was a disturbing figure owing to the widespread distribution of the weed in the environment, and a striking contrast from the previous study by Kapoor where at least 75% of farmers from villages in the Allahabad district of Uttar Pradesh were aware of the same [12]. However, another study by Kapoor as well as by Misra et al. and Neelima et al. reported similar figures of awareness among respondents from Gautam Buddh Nagar district of Uttar Pradesh, Sagar district of Madhya Pradesh, and villages from Andhra Pradesh, respectively, as in the index study [13-15].

More than 70% of our study participants comprised the literate population, with 18% receiving at least a graduate qualification. In this seemingly educated group of respondents, the awareness about the *Parthenium* weed remained low which was consistent with the findings of the above studies where variable awareness was seen irrespective of the level of education of participants [12-15]. Furthermore, despite their education status, almost 60% of our respondents did not know of any methods of control of the spread of *Parthenium*, which causes speculation.

Delving into the ill effects of the weed as the extent of the problem becomes more widespread, there arises an urgent need to look into the methods to get rid of not only the weed but also the prevalent ignorance toward it. As seen in the index and previous studies [12-15], the level of education did not really help with the lacunae in the dispersal of information about the weed. Steps need to be taken toward campaigns to raise awareness through all possible means such as radio/print media/television channels. School-going children may be targeted with information about *Parthenium *in their curriculum that they may pass on to their family and neighborhood. Help may also be obtained from *nukkad nataks* or theaters that capture the public eye.

As most of the respondents could identify the plant, we came across various terminologies that are prevalent for it in the community, which could further help in better communicating with the local population. The study has certain limitations such as the small sample size. A pre- and post-study questionnaire could have been added to assess the change in knowledge of the respondents. These can be undertaken in future studies by the researchers.

## Conclusions

This study was conducted among 250 respondents from a rural community in North India. Despite almost 75% of participants identifying the weed, more than half were neither aware of its ill effects nor knew about the methods of control. Moreover, most of the study population comprised educated individuals yet remained ignorant. Emphasis needs to be placed on awareness campaigns about the fast-spreading *Parthenium *and its ill effects.
